# Is there still a place for ECCO_2_R?

**DOI:** 10.1007/s00063-024-01197-x

**Published:** 2024-10-09

**Authors:** Thomas Staudinger

**Affiliations:** Dept. of Medicine I, Intensive Care Unit, General Hospital of Vienna, Medical University of Vienna, Waehringer Guertel 18–20, 1090 Vienna, Austria

**Keywords:** Respiratory insufficiency, Carbon dioxide, Acute respiratory distress syndrome, Ventilation, Respiration, artificial, Respiratorische Insuffizienz, Kohlenstoffdioxid, Akutes Atemnotsyndrom, Atmung, Künstliche Beatmung

## Abstract

The therapeutic target of extracorporeal carbon dioxide removal (ECCO_2_R) is the elimination of carbon dioxide (CO_2_) from the blood across a gas exchange membrane without influencing oxygenation to a clinically relevant extent. In acute respiratory distress syndrome (ARDS), ECCO_2_R has been used to reduce tidal volume, plateau pressure, and driving pressure (“ultraprotective ventilation”). Despite achieving these goals, no benefits in outcome could be shown. Thus, in ARDS, the use of ECCO_2_R to achieve ultraprotective ventilation can no longer be recommended. Furthermore, ECCO_2_R has also been used to avoid intubation or facilitate weaning in obstructive lung failure as well as to avoid mechanical ventilation in patients during bridging to lung transplantation. Although these goals can be achieved in many patients, the effects on outcome still remain unclear due to lack of evidence. Despite involving less blood flow, smaller cannulas, and smaller gas exchange membranes compared with extracorporeal membrane oxygenation, ECCO_2_R bears a comparable risk of complications, especially bleeding. Trials to define indications and analyze the risk–benefit balance are needed prior to implementation of ECCO_2_R as a standard therapy. Consequently, until then, ECCO_2_R should be used in clinical studies and experienced centers only. This article is freely available.

## Abstract (de)

Das therapeutische Ziel einer extrakorporalen Kohlenstoffdioxid(CO_2_)-Elimination (ECCO_2_R) ist die CO_2_-Entfernung über eine Gasaustauschmembran aus dem Blut, ohne die Oxygenierung relevant zu beeinflussen. Bei Patienten mit „acute respiratory distress syndrome“ (ARDS) können so Tidalvolumen, Plateaudruck und „driving pressure“ gesenkt werden (sog. ultraprotektive Beatmung). Obwohl diese Ziele erreicht werden können, konnten keine positiven Effekte auf Outcomeparameter nachgewiesen werden. Daher kann der Einsatz von ECCO_2_R bei ARDS mit dem Ziel einer ultraprotektiven Beatmung nicht mehr empfohlen werden. Die ECCO_2_R wurde auch eingesetzt, um bei obstruktiven Patienten die die Intubation zu umgehen oder das Weaning zu erleichtern, sowie zur Vermeidung einer Intubation bei hyperkapnischen Patienten im Bridging zur Lungentransplantation. Obwohl diese Ziele bei vielen Patienten erreicht werden können, fehlt Evidenz zu Outcomeparametern. Trotz des geringeren Blutflusses sowie der kleineren Kanülen und Membranen im Vergleich zur extrakorporalen Membranoxygenierung (ECMO) geht ECCO_2_R mit einem vergleichbaren Komplikationsrisiko, vor allem mit Blutungskomplikationen, einher. Studien zur Definition von Indikationen sowie zur Abschätzung des Risiko-Nutzen-Verhältnisses sind dringend nötig, bevor ECCO_2_R als Standardtherapie implementiert werden kann. Daher sollte ECCO_2_R derzeit nur an erfahrenen Zentren und wenn möglich innerhalb klinischer Studien eingesetzt werden. Dieser Artikel ist frei verfügbar.

## Introduction

The therapeutic target of extracorporeal carbon dioxide removal (ECCO_2_R) is the elimination of carbon dioxide (CO_2_) from the blood across a gas exchange membrane without influencing oxygenation to a clinically relevant extent. As ECCO_2_R can be provided by diverse techniques, the term has to be regarded as a therapeutic intention rather than a specific technical procedure [32]. Techniques to achieve the goal of ECCO_2_R include pumpless arteriovenous circuits (pumpless extracorporeal lung assist [PECLA], arteriovenous interventional lung assist [AV-ILA®, Xenios, Heilbronn, Germany]) and low-flow veno-venous circuits based either on the technology of continuous renal replacement therapy (CRRT) or of extracorporeal membrane oxygenation (ECMO).

**Fig. 1:**
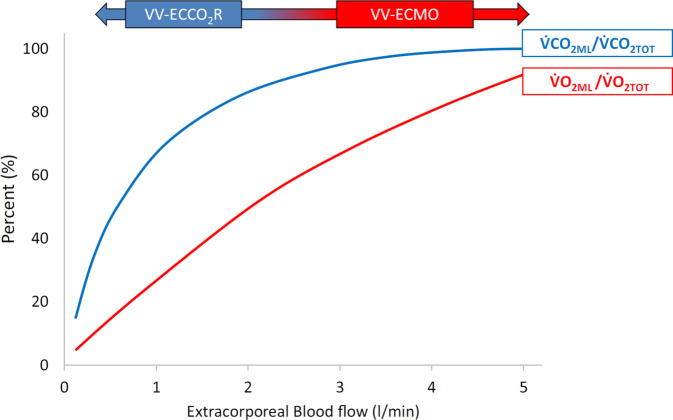
Relationship between extracorporeal blood flow and gas exchange: O_2_ in red and CO_2_ in blue. *VV-ECCO*_*2*_*R* veno-venous extracorporeal carbon dioxide removal, *VV-ECMO* veno-venous extracorporeal membrane oxygenation. (From [35], © T. Tonetti et al., CC BY 4.0; https://creativecommons.org/licenses/by/4.0/)

In contrast to oxygen, CO_2_ transport in blood consists mainly of bicarbonate, which is converted into CO_2_ and water by carbonic anhydrase and less than 5% is bound to hemoglobin. Due to its greater solubility, CO_2_ diffuses more readily than oxygen across a gas exchange membrane [28] Therefore, blood decarboxylation is a more efficient, non-limited process compared with oxygenation and can be achieved at blood flow rates much lower than necessary for clinically relevant oxygenation (Fig. 1; [7]). The efficiency of extracorporeal decarboxylation depends mainly on the membrane surface, inlet PaCO_2_, sweep gas flow, and blood flow. Because theoretically the whole amount of CO_2_ produced can be cleared from 1 L of blood, in contrast to oxygenation, increasing blood flow to more than 1000–2000 mL/min will not increase CO_2_ removal to substantially higher amounts [15, 22].

**Fig. 2:**
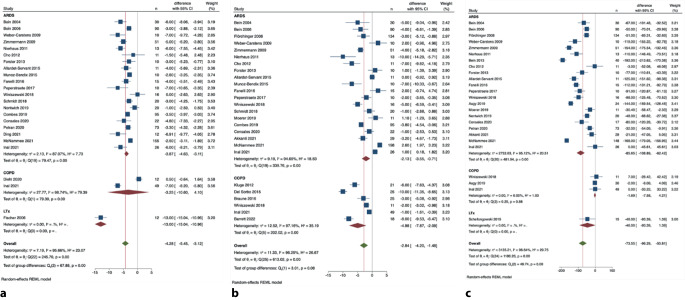
Change in plateau pressure (**a**), respiratory rate (**b**), and tidal volume (**c**) within 24 h of initiating ECCO_2_R according to diagnosis subgroups. *ARDS* acute respiratory distress syndrome, *COPD* chronic obstructive pulmonary disease, *LTx* lung transplantation. (From [33], © A.-M. Stommel et al., CC BY 4.0; https://creativecommons.org/licenses/by/4.0/)

The first clinical use of “ECCO_2_R” achieved by low-flow veno-venous extracorporeal gas exchange was in 1986 by Gattinoni’s group in Milan to reduce ventilator-associated lung injury in 43 patients with severe acute respiratory distress syndrome (ARDS; [20]). In the following years, the concept was abandoned in favor of veno-venous (VV)-ECMO, additionally providing oxygenation. During the period 2000–2010, the development of the pumpless arteriovenous PECLA (also called ILA®—Interventional Lung Assist®, Novalung, Heilbronn, Germany) led to a renaissance of the concept of using ECCO_2_R in ARDS to enhance gas exchange as well as to avoid ventilator-induced lung injury by achieving less invasive ventilator settings [5]. In the ensuing years, to overcome the disadvantage of arterial cannulation, a number of pump-driven veno-venous devices for ECCO_2_R entered the market, either based on roller pump-driven CRRT technology or centrifugal pump-driven minimized ECMO systems [32]. Together with the evolving concept of “ultraprotective ventilation” in ARDS, the technological innovations initiated all but a boom in ECCO_2_R as an adjunct to mechanical ventilation for avoiding ventilator-induced lung injury (Fig. 2). Moreover, ECCO_2_R has been used to avoid intubation or to facilitate ventilation and weaning in obstructive lung failure, mainly acute exacerbated chronic obstructive pulmonary disease (AECOPD), as well as to avoid mechanical ventilation in patients during bridging to lung transplantation (LTX).

## ECCO_2_R and ARDS

Based on the observation that increasing plateau pressure correlates positively with mortality in ARDS patients [21], the assumption that decreasing tidal volume below 6 mL/kg predicted body weight (PBW)—a “dogma” for ARDS patients since the ARMA trial [2]—would lead to even less lung injury and thus improve outcome further seems a reasonable one. This concept is known as “ultraprotective” ventilation. In 32 ventilated ARDS patients, Terragni et al. showed a decrease in pro-inflammatory cytokines in the broncho-alveolar lavage fluid, as a surrogate for ventilator-induced lung injury, when tidal volume (and thus plateau pressure) was reduced to levels below standard protective ventilation [34]. In this study, the goal of ultraprotective ventilation was achieved by concomitant ECCO_2_R using a CRRT-based system equipped with a gas exchange membrane instead of a hemofilter.

In the following years, a considerable number of retrospective and prospective cohort studies proved that achieving ultraprotective ventilation in ARDS patients, resembling tidal volumes between 3 and 4 mL/kg PBW, is feasible by different ECCO_2_R methods. The largest of these cohort studies, the SUPERNOVA trial, reported on a significant reduction in tidal volume and plateau pressure, concomitantly preserving normocapnia and oxygenation after initiation of different ECCO_2_R systems [12]. Of note, the so-called higher-extraction systems (more efficient CO_2_ removal by higher blood flow and larger membranes) were more effective [14]. Recently, a comprehensive systematic review and meta-analysis by our group analyzing the pooled data from all cohort studies published to date confirmed these findings (Fig. 1; [33]). The question remains, however, whether ultraprotective ventilation translates into improved outcome.

Only two prospective randomized trials to compare standard protective versus ultraprotective ventilation have been conducted so far: The first one, the XTRAVENT trial, had to be terminated prematurely due to enrolment problems. ECCO_2_R plus ultraprotective ventilation with tidal volumes as low as 3 mL/kg PBW in patients with mild to moderate ARDS did not lead to significantly shorter time on the ventilator or in the intensive care unit or to improved survival. In the subgroup with a PaO_2_/FiO_2_ ratio of < 150, however, patients could be weaned off mechanical ventilation faster [4]. A much larger multicenter, randomized trial (REST trial) with more or less the same design was published 8 years later [29]. The results were likewise disappointing: Despite a significant reduction in tidal volume, plateau pressure, and driving pressure in the ECCO_2_R group, no significant difference with respect to mortality was detected. Mortality tended to be even higher in the intervention group. A considerable number of device-related serious side effects were reported, which might have blocked the possible beneficial effects of ECCO_2_R. Thus, to date, there is no evidence supporting the concept of ultraprotective ventilation enabled by ECCO_2_R to improve outcome.

### Open questions.

The use of ECCO_2_R in patients with mild to moderate ARDS independent of the concept of ultraprotective ventilation has not been investigated so far. In other words, patients in whom standard protective ventilation settings cannot be achieved due to predominant hypercapnia and respiratory acidosis could hypothetically benefit from CO_2_ removal. According to current recommendations, however, refractory respiratory acidosis resembles an indication for VV-ECMO. In severe hypoxic ARDS, VV-ECMO has been shown to improve outcome [13]. It remains unclear whether this also holds true for ARDS patients suffering predominantly from hypercapnia with less impaired oxygenation or whether ECCO_2_R might bring about advantages in these situations. The complication rates seem comparable, but it is also not clear whether different technologies for ECCO_2_R differ with respect to complications (see below).

## ECCO_2_R and COPD/asthma

Patients with severe AECOPD and consecutive failure of noninvasive ventilation (NIV) leading to mechanical ventilation have a poor prognosis [11]. ECCO_2_R has been shown to effectively avoid mechanical ventilation in patients at high risk for NIV failure: A cohort study of AECOPD patients with emerging NIV failure showed that by using pumpless arteriovenous ECCO_2_R, intubation could be avoided in 90% of cases [27]. In another similar study using a miniaturized veno-venous axial pump-driven ECCO_2_R system, 56% of patients could be managed without intubation [8]. Complications, especially bleeding events, occurred in a considerably high proportion of patients (44%). Table 1 summarizes the small studies conducted to date involving the use of ECCO_2_R in patients with AECOPD to avoid intubation. Three studies compared ECCO_2_R patients with matched controls and found no major differences with respect to outcome, except for the study by Del Sorbo et al., who reported on a higher intubation rate and mortality in the control group [16]. A small prospective randomized trial did not find any differences in outcome parameters, except for a prolonged length of stay in the ECCO_2_R group [3].

**Table 1:** Studies on ECCO_2_R in patients with AECOPD to avoid intubation

Study/*n*	ECCO_2_R			Outcome		Comment
	Device	Blood flow (mL/min)	Duration	Intubation rate	Mortality (hospital)	
Cohort [27] *n* = 21	AV-ILA©	1100 (600–1800)	9 (1–116) d	10%	24% (28–d)	Matched control group (*n* = 21; intubated after NIV failure): No significant difference in mortality and LOS
Cohort [10] *n* = 7	Hemolung©	431 ± 74	104 ± 60 h	0	57%	–
Cohort [16] *n* = 25	Decap Smart©	255 ± 78	29 ± 5 h	12%	8%	Matched control group (*n* = 21; NIV without ECCO_2_R): Significantly higher intubation rate (33%) and mortality (35%)
Cohort [8] *n* = 25	ILA Activve©	1300 (700–1800)	8.5 (1.0–27.0) d	44%	24%	Matched control group (*n* = 25; intubated after NIV failure): No significant difference in mortality and LOS
RCT [3] *n* = 9 (NIV) *n* = 9 (NIV + ECCO_2_R)	Hemolung©	400 (365–410)	96 (60–138) h	0 in both groups	89% (NIV) 67% (NIV + ECCO_2_R)	LOS significantly longer with ECCO_2_R

*AECOPD* acute exacerbated chronic obstructive pulmonary disease, *d* days, *ECCO*_*2*_*R* extracorporeal carbon dioxide removal, *h* hours, *LOS* length of stay, *NIV* noninvasive ventilation

ECCO_2_R might also have positive effects in mechanically ventilated COPD patients to enhance weaning. Data are sparse, yet a few small observational studies report on weaning rates between 30% and 100% in this setting [1, 10, 26].

Severe refractory asthma might also be a possible indication for ECCO_2_R. Aside from a few case reports, the largest study so far reports on 26 patients with status asthmaticus undergoing extracorporeal CO_2_ removal, leading to a survival rate of 100% [9]. However, “ECCO_2_R” in this study was performed in a veno-venous, two-cannula setting with a mean blood flow of 3.2 L/min, thus more or less resembling VV-ECMO. The beneficial role of VV-ECMO in refractory status asthmaticus is well established [17, 36], while evidence for “exclusive” CO_2_-removing low-flow systems is sparse.

### Open questions.

By removing CO_2_ from blood, ECCO_2_R is able to reduce respiratory drive and respiratory rate, thus prolonging expiration time and thereby—theoretically—enabling reduction of overinflation in patients with obstructive pulmonary disease. “High extraction” systems seem somewhat more effective to reduce respiratory rate [33]. The lack of evidence with respect to outcome and the balance between possible beneficial effects and complications means the therapy is still an experimental one. Larger prospective controlled trials on intubation rate and early weaning as well as outcome in COPD patients at risk for NIV failure or on mechanical ventilation are urgently warranted before implementing ECCO_2_R as a standard therapy in patients with obstructive pulmonary disease.

## ECCO_2_R and lung transplantation

Respiratory failure during bridging to LTX necessitating intubation and mechanical ventilation leads to significant mortality and less successful outcome after transplantation. In this situation, keeping patients awake, breathing spontaneously, and ambulant can be achieved by extracorporeal gas exchange, either ECMO or ECCO_2_R [6, 19, 23]. Many of these patients, especially those with terminal fibrosis, suffer from predominant hypercapnia. ECCO_2_R has been successfully used for these patients as a bridge to LTX (Table 2; [18, 30, 31]).

**Table 2:** Studies on the use of ECCO_2_R as a bridge to LTX

Study/*n*	Device(s)	Bridging time (days)	LTX	Hospital survival
Cohort [18] *n* = 12	AV-ILA®	15 ± 8	10 (83%)	8 (67%)
Cohort [30] *n* = 12	AV-ILA® (*n* = 6) Decap Smart® (*n* = 6)	13.5 ± 14.2	3 (25%)	4 (33%)
Cohort [31] *n* = 20	AV-ILA® (*n* = 10) ILA Activve® (*n* = 10)	8 (4–11)	19 (95%)	15 (75%)

*ECCO*_*2*_*R* extracorporeal carbon dioxide removal, *LTX* lung transplantation

### Open questions.

As ambulation seems to be a major factor for successful bridging, less invasive systems avoiding cannulation of the groin may bring about advantages. However, this can be also achieved by double-lumen ECMO cannulas. It remains unclear whether low-flow systems offer advantages over VV-ECMO with respect to management and complications.

## ECCO_2_R and complications

Less blood flow, smaller cannulas, and smaller gas exchange membranes do not automatically translate into a lower complication rate. Arterial cannulation necessary for pumpless ECCO_2_R (PECLA, AV-ILA®) may lead to limb ischemia, while in pump-driven veno-venous low-flow systems bleeding and mechanical complications are predominant. Table 3 lists the rate of clinically relevant complications of ECCO_2_R as reported in the literature and summarized in a systematic review [33]. No major differences in complication rate between low- and high-extraction systems were observed. The reasons for the considerably high rate of bleeding complications may be anticoagulation but also the increasing blood trauma by centrifugal pumps originally designed for high-flow systems (ECMO), especially if low blood volume is processed [25]. The largest randomized trial on ECCO_2_R in ARDS to date reported on 62 (31%) patients in the intervention group experiencing serious adverse events compared with 18 (9%) in the standard care group. Overall, 22 events (11%) were categorized as related to ECCO_2_R, including five intracranial bleeding events. Most bleeding events were described when centrifugal or axial pump-driven technologies were used. It remains unclear whether CRRT-based systems, either in combination with renal replacement therapy or as stand-alone systems, result in comparably high complication rates, since the patient numbers treated by those systems are reported to be much lower. The advantage of regional anticoagulation by citrate, however, does not apply to CRRT-based ECCO_2_R, as blood flows of 300–400 mL/min are needed to achieve clinically relevant decarboxylation (see also Fig. 1).

**Table 3:** Reported pooled rates of adverse events [33]

			Clinically significant		
	Sample size	All adverse events	Bleeding events	Thrombotic or ischemic events	Technical adverse events
***High-extraction systems***					
Subgroup pooled rate (95% CI)	566	0.21 (0.14–0.32)	0.04 (0.02–0.08)	0.09 (0.05–0.16)	0.02 (0.01–0.04)
***Low-extraction systems***					
Subgroup pooled rate (95% CI)	799	0.10 (0.04–0.25)	0.05 (0.02–0.12)	0.03 (0.01–0.08)	0.03 (0.01–0.06)
***Mixed use of systems***					
*Subgroup pooled rate (95% CI)*	186	0.77 (0.57–1.00)	0.14 (0.06–0.33)	0.17 (0.12–0.24)	0.07 (0.03–0.15)
**Overall pooled rate (95% CI)**	1551	0.19 (0.12–0.28)	0.05 (0.03–0.08)	0.07 (0.04–0.11)	0.02 (0.01–0.04)

## Conclusion

After more than 20 years of “modern” extracorporeal carbon dioxide removal (ECCO_2_R), no evidence with respect to improved outcome exists. Therefore, ECCO_2_R still has to be regarded as experimental therapy and bears the risk of major complications. However, ECCO_2_R is an effective therapy to settle respiratory acidosis, thus economizing respiratory mechanics either in mechanically ventilated or in spontaneously breathing patients [33]. It can indeed be impressive to watch a near-comatose hypercapnic patient awaking and breathing more and more calmly and economically, and thus being able to move and even walk without the need for mechanical ventilation. Unfortunately, reliable data on these specific situations are missing.

In acute respiratory distress syndrome, the use of ECCO_2_R to achieve ultraprotective ventilation can no longer be recommended. Many companies and manufacturers have therefore left the market and most technologies are no longer available. Severely hypercapnic patients with chronic obstructive pulmonary disease or terminal fibrosis who have the option of lung transplantation seem to be a more rewarding target for ECCO_2_R, yet outcome-targeted trials are missing. Consequently, ECCO_2_R should be applied at experienced centers only, ideally in clinical studies. Moreover, trials on new, optimized ECCO_2_R systems based on continuous renal replacement therapy, which might be nearly as effective as centrifugal pump-driven “high-extraction” systems [24], are needed to reliably estimate the balance between possible clinical benefits and concomitant risks.

## Declarations

For this article no studies with human participants or animals were performed by any of the authors. All studies mentioned were in accordance with the ethical standards indicated in each case.

The supplement containing this article is not sponsored by industry.
